# Nrf2-Mediated Antioxidant Activity of the inner bark extracts obtained from *Tabebuia rosea* (Bertol) DC and *Tabebuia chrysantha* (JACQ) G. Nicholson.

**DOI:** 10.12688/f1000research.17165.2

**Published:** 2019-02-12

**Authors:** Sandra C. Garzón-Castaño, Iván A. Lopera-Castrillón, Francisco J. Jiménez-González, Fernando Siller-López, Luz A. Veloza, Juan Carlos Sepúlveda-Arias

**Affiliations:** 1 Grupo Infección e Inmunidad, Facultad de Ciencias de la Salud, Universidad Tecnológica de Pereira, Pereira, Risaralda, 660003, Colombia; 2 Grupo de Biomedicina, Facultad de Medicina, Fundación Universitaria Autónoma de las Américas, Pereira, Risarala, 660003, Colombia; 3 Grupo Polifenoles, Facultad de Tecnologías, Universidad Tecnológica de Pereira, Pereira, Risaralda, 660003, Colombia

**Keywords:** Tabebuia chrysantha, Tabebuia rosea, Bignoniaceae, extracts, Nrf2, antioxidant agents.

## Abstract

**Background: **Several ethnobotanical and ethnopharmacological studies have shown the therapeutic potential of plants from the genus *Tabebuia*, which have long been used in traditional medicine in rural areas of South America, for the treatment of several human diseases. This study aimed to evaluate the Nrf2-mediated antioxidant activity of the inner bark extracts obtained from *Tabebuia rosea* and *Tabebuia chrysantha*.

**Methods: **The antioxidant activity of extracts obtained from the inner bark of *T. rosea* and *T. chrysantha* was evaluated using the Oxygen radical absorbance capacity (ORAC) technique. The effect of extracts on the viability of HepG2 cells was determined using the 3-(4,5-dimethylthiazol-2-yl)-2,5-diphenyl tetrazolium bromide (MTT) method. The translocation of Nrf2 to the nucleus after exposure of HepG2 cells to the extracts and controls (α-lipoic acid, curcumin and hydrogen peroxide) was evaluated using the Nrf2 transcription factor kit. Induction of the Nrf2-mediated antioxidant response gene ( *NQO1*) was evaluated by real-time PCR.

**Results: **The ethyl acetate extract obtained from both species displayed the highest ORAC activity (12,523 and 6,325 µmoles Eq Trolox/g extract). In addition, the extracts had the ability to activate and to translocate Nrf2 to the nucleus, as well as to induce the expression of *NQO1*.

**Conclusion:** These results indicate that the ethyl acetate extracts obtained from the inner bark of *T. chrysantha* and *T. rosea* have an important antioxidant effect mediated by Nrf2 activation, and could be used as a new source of natural antioxidants.

**Abbreviations used:** AAPH: 2,2’-Azobis (2-amidinopropane) dihydrochloride; ALA: Alpha-lipoic acid; CUR: Curcumin; ORAC: Oxygen radical absorbance capacity; MTT: 3-(4,5-dimethylthiazol-2-yl)-2,5-diphenyl tetrazolium bromide.

## Introduction

Nature’s compounds reveal a great diversity of chemical structures and physicochemical properties, as well as biological ones. Over the years, plants have been used for the treatment of various diseases, including those of inflammatory origin, such as arthritis, obesity, and cancer. Plants of the genus *Tabebuia* belong to the Bignoniaceae family, which is composed of about 120 genera with 827 species, and is considered the second most diverse family of species of neotropical woody plants in dry forests ^1^. There are reports on the presence of chemical compounds⎯including quinones and phenols, among others⎯in this family ^2, 3^. The *Tabebuia* genus comprises about 100 species of trees and shrubs, mainly distributed from Mexico to several regions of Central and South America, which have been used in traditional medicine. Plants of this genus are an important source of bioactive molecules such as: naphthoquinones; quinones; phenols; and molecules with anti-inflammatory, antioxidant, anti-microbial and anti-proliferative activity ^4– 8^.

A large number of chemical compounds exert their antioxidant effects through the activation of key transcriptional regulation mechanisms, such as the transcription factor Nrf2 (nuclear factor erythroid 2-related factor 2) ^9^. Under normal physiological conditions, this factor is in the cytoplasm inhibited by Keap1 (Kelch ECH associating protein 1), which leads to its degradation ^10^. In cells exposed to oxidative stress, Nrf2 is not targeted for ubiquitin-dependent degradation. Instead, it is released and translocated to the nucleus, where it activates its antioxidant response through binding to Antioxidant Response Elements (ARE sites), allowing the coordinated expression of more than 200 detoxifying enzymes and antioxidants, such as NAD(P)H quinone oxidoreductase (NQO1) and heme oxygenase 1 (HO-1), among others ^11– 14^. The aim of this study was to evaluate the Nrf2-mediated antioxidant activity of extracts obtained from the inner bark of *Tabebuia rosea* and *Tabebuia chrysantha* in HepG2 cells.

## Methods

### Plant material and extract preparation

The inner bark from *T. rosea* (Bertol.) DC and *T. chrysantha* (JACQ) G. Nicholson were collected at Universidad Tecnológica de Pereira campus in April 2011. The plants were identified at the Colombian National Herbarium (Voucher No. COL 582577 for *T. rosea* and COL 587611 for *T. chrysantha*). The collection and processing of the material were covered by the collection permission number 1133/2014, issued by the National Environmental Licensing Authority (ANLA) of Colombia.

Extracts were obtained as previously described ^6^. Plant material (inner bark from *T. chrysantha* and *T. rosea*) was dried and macerated in methanol analytical grade (14 L) for 48 h. This was followed by homogenization, filtration, and concentration under vacuum using a vacuum rotary evaporator (Heidolph, Laborota model) at 40 °C to obtain the crude extract. This procedure was repeated three times. Crude extracts were dissolved in 400 mL of distilled water, and underwent liquid–liquid extraction with increasing polarity solvents (solvent volume 1.6 L): *n*-hexane, chloroform (CHCl _3_), ethyl acetate (EtOAc), and *n*-butanol (all solvents were analytical grade). Each extract was vacuum dried by vacuum rotary evaporator obtaining the following mass for each extract: *n*-hexane (0.3 g), chloroform (1.2 g), ethyl acetate (3.7 g), *n*-butanol (12.5 g) and aqueous (21.7 g). Endotoxin levels in the extracts were assayed using the Limulus Amebocyte Lysate Test, E-Toxate Kit (Sigma Chemical Co, Saint Louis, MO, USA, Cat No. ET0200-1KT). All samples were negative for the presence of endotoxins (detection limit 0.05–0.1 EU/mL). The extracts were kept refrigerated at 4 °C in amber tubes protected from light, heat, air and moisture. For each one of the biological assays, the extracts were dissolved in 0.1% DMSO (Dimethyl sulfoxide, Merck, Darmstadt, Germany, Cat No. 1029521000).

### Preliminary phytochemical screening

The preliminary phytochemical screening was performed using selective derivatization reactions for the characterization of secondary metabolites present in the *n*-hexane, chloroform, ethyl acetate and *n*-butanol extracts obtained from inner bark, as previously reported for *T. rosea* ^6^. The extracts were evaluated using normal and reverse phase thin layer chromatography (TLC). Chromatographic plates were revealed with aluminum chloride (AlCl _3_, Sigma Chemical Co, Saint Louis, MO, USA, Cat No. 563919-25G) and ferric chloride (FeCl _3_, Sigma Chemical Co, Saint Louis, MO, USA, Cat No. 157740-1KG) for detection of flavonoids, phenols and phenolic acids; potassium hydroxide (KOH, Merck, Darmstadt, Germany, Cat No. 1050331000) in analytical grade ethanol for detection of anthrones, quinones and coumarins; oleum (Sigma Chemical Co, Saint Louis, MO, USA, Cat No. 778990-500ML) for detection of sesquiterpenic lactones; and the Liebermann-Burchard reagent for detection of terpenes and steroids.

### Total phenolic content

The total phenolic content of each extract was determined according to the Folin–Ciocalteu colorimetric method ^15^, using gallic acid as standard. Briefly, Folin-Ciocalteu’s reactive (Merck, Darmstadt, Germany, Cat No. 1090010100) was diluted 10-fold with distilled water. 25 µL of the samples (1 mg/mL) were added to the Folin-Ciocalteu’s reactive. After the addition of Na _2_CO _3_ (20%), the reaction was maintained at room temperature (RT) in the dark for 30 min, and absorbance was measured at 760 nm in a Shimadzu UV-1700 spectrophotometer. Gallic acid (0.25–5 mg/mL) was used to generate a standard curve (y=0.101x+0.086; *R* ^2^=0.996). Results are presented as mg gallic acid equivalents per g of extract (mg GAE/g extract). All experiments were performed in triplicate.

### Oxygen radical absorbance capacity (ORAC)

Oxygen radical absorbance capacity was determined using the method described by Ou *et al*, with some modifications ^16^. 2,2’-Azobis (2-amidinopropane) dihydrochloride (AAPH) and sodium fluorescein stock solutions were prepared in a 75 mM, pH 7.0 phosphate buffer solution. 31 μL of each sample were diluted in 187 μL of fluorescein (120 nM) and incubated at 37 °C for 10 min. The reaction was started by the addition of 31 μL of AAPH (143 mM) to reach a final volume of 249 μL per well. Extracts were evaluated at 10, 15, and 20 μg/mL. A Trolox® standard curve was prepared (10, 20, 40, and 60 μM). Changes in fluorescence were measured with a Varian Cary Eclipse 1.1 fluorescence spectrophotometer at 2 min intervals for 120 min with emission and excitation wavelengths of 515 and 493 nm, respectively. The excitation slit was 5 nm, as was the emission slit ^17, 18^. The antioxidant capacity was calculated as the area under the curve (AUC) ^19^ and expressed as μmol Trolox® equivalents per g of extract (μmol TE/g of extract).

### Cell culture

Human hepatocarcinoma cell line (HepG2; ATCC; CRL-11997) was purchased from American Type Culture Collection (ATCC, Rockville, MD, USA) and cultured with Dulbecco’s Modified Eagle Medium (DMEM), supplemented with Glutamax (GIBCO/BRL, USA, Cat No. 10564-011) and 10% heat inactivated FBS (GIBCO, Cat No. 16140071), 200 μg/mL Penicillin, 200 μg/mL streptomycin, 400 μg/mL neomycin (GIBCO, Cat No. 15640-055), 5 μg/mL amphotericin, 0.05 mM 2-β-mercaptoethanol, and 1 mM sodium pyruvate (all from Sigma Chemical Co, Saint Louis, MO, USA, Cat No. A9528-50MG, M7522-100ML, S8636-100ML, respectively). Cells were maintained at 37 °C with 5% CO _2_ atmosphere.

### Cell viability test

To determine the effects of extracts on HepG2 cells, cell viability was tested using the MTT (3-(4,5-dimethylthiazol-2-yl)-2,5-diphenyl tetrazolium bromide) method ^20^. Cells (5 × 10 ^4^ cell/well) were treated with a concentration of 2 μg/mL of the extracts (the *n*-butanol extract from *T. chrysantha* was used at 1 μg/mL) diluted in DMSO (final concentration 0.1%), and incubated for 24 hours. After treatment, the medium was discarded, 200 μL of DMEM medium containing 0.5 mg/mL MTT (Sigma Chemical Co, Saint Louis, MO, USA, Cat No. M2128-500MG) was then added to each well. The plates were incubated for 4 hours at 37°C in a 5% CO _2_ atmosphere. The medium was discarded and 100 μL of DMSO was then added to solubilize the formazan crystals. Absorbance was measured in an ELISA microplate reader at 492 nm (ELx800; BioTek Instruments Inc., USA). Viability percentage was calculated based on the non-treated control. Three independent assays were performed, each one in triplicate.

### Nrf2 nuclear activation

The HepG2 cell line (3 × 10 ^5^ cell/well) was cultured in DMEM medium using a T25 flask. The medium was discarded, and the cells were exposed at two time points (0 and 4 hours) to: Alpha-lipoic acid (ALA, 50 μM, Sigma Chemical Co, Saint Louis, MO, USA, Cat No. T1395-1G), Curcumin (CUR, 15 μM, Sigma Chemical Co, Saint Louis, MO, USA, Cat No. C7727-500MG) ^21– 24^, ethyl acetate extracts from *T. chrysantha* (0.5 μg/mL), ethyl acetate extract from *T. rosea* (1 μg/mL) and H _2_O _2_ (0.6 mM). H _2_O _2_ was used as an oxidative stress inductor. After exposure, cells were harvested and used for nuclear and cytosolic protein extraction simultaneously, following the specifications included in the Nuclear Extraction Kit (Cayman Chemical, Ann Arbor MI, USA, Item No 10009277). Protein fractions were quantified using the BCA Protein Quantification Kit (Abcam, Cambridge, UK, ab102536). Nrf2 was detected by using the Nrf2 transcription factor assay kit (Cayman Chemical, Ann Arbor MI, USA, Item No 600590), and following the manufacturer’s instructions. The absorbance of each well was measured at 450 nm in an ELx800 BioTek microplate reader.

### qRT-PCR assays

HepG2 cells (3 × 10 ^5^ cells/well) were treated with ALA (50 μM), CUR (15 μM, ethyl acetate extract from *T. chrysantha* (0.5 μg/mL), ethyl acetate extract from *T. rosea* (1 μg/mL) and H _2_O _2_ (0.6 mM) for durations of 0, 6 and 12 hours. After treatment, mRNA extraction was performed using the RNeasy Plus Mini Kit (Qiagen, Maryland, USA, Cat No. 74134). The mRNA was quantified with a NanoDrop 2000c (Thermo Fisher Scientific, Waltham, MA). The expression of the *NQO1* gene (an Nrf2-dependent gene containing ARE sequences in its promoter region ^25, 26^ that is expressed in response to oxidative stress) was evaluated by qRT-PCR and quantified using the 2 ^-ΔΔCt^ method, using predesigned TaqMan Gene Expression Assays (code Hs00168547, Applied Biosystems, Foster City, CA, Cat No. 4331182) and the TaqMan® RNA-to-CT ^TM^ 1-Step kit (Applied Biosystems, Foster City, CA, Cat No. 4392653). The run method was holding 48 °C and 15 min, 95 °C 10 min and cycling (40 cycles) 95 °C 15 sec, 60 °C 1 min. β-actin was used as an endogenous control.

### Statistical analysis

Each experiment was performed at least in duplicate. Results were expressed as the mean ± SD of at least three independent experiments. Statistical analysis was performed using the Kruskal-Wallis test and a *p*-value less than 0.05 was considered statistically significant. The statistical tests were applied using GraphPad Prism, version 5.02 (GraphPad Software, San Diego, CA, USA).

## Results

### Preliminary phytochemical screening, total phenolic content, ORAC and cell viability

The preliminary phytochemical screening of the inner bark extracts obtained from *T. rosea* and *T. chrysantha* did show the presence of anthrones, quinones and coumarins, as previously reported for *T. rosea* ^6^ ( Table 1). Sesquiterpenic lactones were present in the *n*-hexane, chloroform and ethyl acetate extracts of *T. rosea*, but were absent from the *n*-hexane extract from *T. chrysantha*. Steroids were identified in chloroform and ethyl acetate extracts of both species. The presence of flavonoids and phenolic acids was observed only in the ethyl acetate extract from *T. rosea*. The total phenolic content in the extracts was determined by the Folin Ciocalteu colorimetric method. The ethyl acetate extracts obtained from *T. rosea* and *T. chrysantha* exhibited the highest total phenolic content (2.18 ± 0.29 and 2.08 ± 0.72 mg GAE/g extract, respectively), whereas the chloroform and aqueous extracts displayed the lowest phenolic content (0.63 ± 0.11, 1.55 ± 0.78, -0.668 ± 0.23 and 0.07 ± 0.03 mg GAE/g extract, respectively). The total phenolic content of the ethyl acetate extract from *T. rosea* was significantly higher ( *p* <0.05) than its chloroform extract. The order of total phenolic content in both species is: ethyl acetate> *n*-butanol> chloroform> aqueous ( Table 2). The ORAC results indicated that the ethyl acetate extracts from *T. rosea*, at a concentration of 10 µg/mL, have the best antioxidant activity (12,523.41 ± 840.46 µmol TE/g extract) and even this activity was superior to that obtained with the controls, showing a significant difference ( *p*<0.001) compared to the antioxidant activity of quercetin ( Table 2). Both the chloroform and *n*-butanol extracts from *T. rosea* also showed important activity. Among the *T. chrysantha* extracts, the ethyl acetate extract displayed the best antioxidant activity (6,325.74 ± 1,057.14 µmol TE/g extract); however, this activity did not exceed that obtained with luteolin and quercetin ( Table 2). The MTT assay revealed that neither the extracts from the inner bark of *T. rosea* nor those from *T. chrysantha* affected the viability of the cells studied, since viability was greater than 80% after 24 hours of exposure ( Table 2).

**Table 1.** Preliminary phytochemical screening of extracts obtained from the inner bark of *T. rosea* and *T. chrysantha*.

Part of the plant	Extract	Reagent				
		AlCl _3_	KOH/EtOH	Oleum	FeCl _3_	Liebermann-Burchard
**Inner bark** ***T. rosea***	*n*-Hexane CHCl _3_ EtOAc *n*-Butanol	**-**	**+**	**+**	**-**	**+**
		**-**	**+**	**+**	**-**	**+**
		**-**	**+**	**+**	**+**	**+**
		**-**	**+**	**-**	**-**	**-**
**Inner bark** ***T. chrysantha***	*n*-Hexane CHCl _3_ EtOAc *n*-Butanol	**-**	**-**	**-**	**-**	**-**
		**+**	**+**	**+**	**-**	**+**
		**+**	**+**	**+**	**-**	**+**
		**+**	**+**	**-**	**+**	**-**

+: Presence of compounds; -: Absence of compounds; KOH: Potassium hydroxide; AlCl _3_: Aluminum chloride; FeCl _3_: Ferric chloride.

**Table 2.** Total phenolic content, ORAC activity and cell viability in extracts obtained from the inner bark obtained from *T. chrysantha* and *T. rosea*.

Extract		Phenolic content (mg GAE/g extract)	ORAC (µmol TE/g extract)	Cell viability (%)
***T. rosea***	n-Hexane	nd	1,631.13 ± 285.52	90.55 ± 5.62
	CHCl _3_	0.63 ± 0.11	5,794.01 ± 586.42	90.77 ± 6.05
	EtOAc	2.18 ± 0.29 ^a^	12,523.41 ± 840.46 ^b^	93.64 ± 5.88
	*n*-Butanol	0.91 ± 0.10	7,539.69 ± 851.01	95 ± 7.47
	H _2_O	-0.66 ± 0.23	1,236.89 ± 332.45	92.33 ± 4.97
***T. chrysantha***	*n*-Hexane	nd	237.79 ± 402.29	89.28 ± 14.9
	CHCl _3_	1.55 ± 0.78	4,124.98 ± 474.52	96.05 ± 7.52
	EtOAc	2.08 ± 0.72	6,325.74 ± 1,057.14	91.99 ± 8.13
	*n*-Butanol	2.01 ± 0.18	5,103.54 ± 1,151.73	89.66 ± 3.18
	H _2_O	0.07 ± 0.03	475.56 ± 498.68	94.16 ± 8.82
**Control**	Luteolin	nd	10,426.71 ± 2,761.28	nd
	Quercetin	nd	8,175.36 ± 444.86	nd

TE: Trolox equivalents; GAE: Gallic acid equivalents; nd: Not determined. All experiments were carried out in triplicate, and the data represent the mean ± SD. Kruskal Wallis ^a^ *p*<0.05 compared to CHCl _3_ from *T. rosea*, ^b^ *p*<0.001 compared to quercetin.

### Effect of *T. rosea* and *T. chrysantha* extracts on activation and nuclear translocation of Nrf2

**Figure 1.**
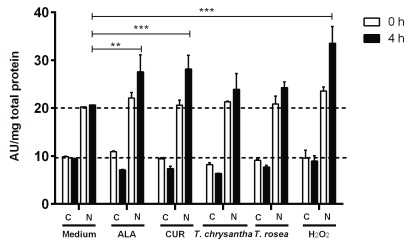
Nrf2 levels in cytosol (C) and nucleus (N) after 0 (white bars) and 4 hours (black bars) exposure to 50 mM ALA, 15 mM CUR, ethyl acetate extracts from *T. chrysantha* (0.5 μg/mL) and *T. rosea* (1 µg/mL) and induction of oxidative stress with 0.6 mM H _2_O _2_. Kruskal Wallis ** *p*<0.01, *** *p*<0.001. ALA: Alpha-lipoic acid; CUR: Curcumin.

The Nrf2 detection test allowed for the evaluation of the ability of the extracts to activate and translocate Nrf2 to the nucleus. Nrf2 detection enabled comparisons of the basal Nrf2 status in both the cytosol and the nucleus. It also allowed for comparison of their associated changes after the exposure of HepG2 cells to the ethyl acetate extracts from *T. chrysantha* (0.5 μg/mL) and *T. rosea* (1 μg/mL), which displayed the best antioxidant activity in the ORAC assay. As shown in Figure 1, the exposure of HepG2 cells to ALA, CUR, H _2_O _2_, and the ethyl acetate extract from both *T. chrysantha* and *T. rosea*, decreased the Nrf2 levels in the cytoplasm after 4 hours of exposure, although the differences are not significant. This decrease was measured in relation to their basal level (non-exposed cells). As expected, an increase in Nrf2 levels in the nucleus was observed after exposure to ALA, CUR, H _2_O _2_ and the extracts. However, significant differences were found only after exposure to ALA, CUR and, H _2_O _2_ ( *p*<0.01).

### Effect of extracts on the expression of *NQO1*

**Figure 2.**
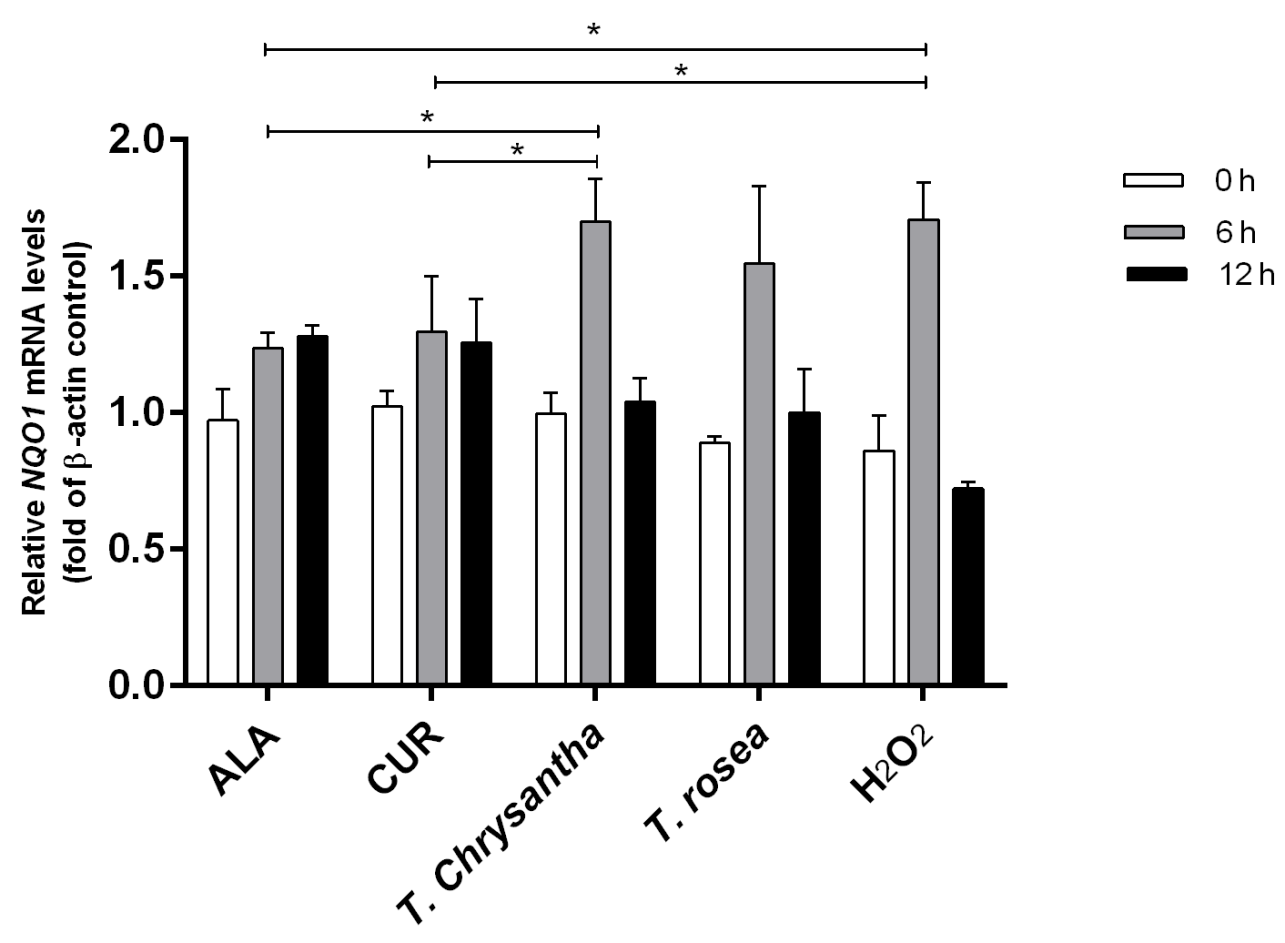
Relative *NQO1* mRNA levels with 0, 6 and 12 hours post-exposure to 50 mM ALA, 15 mM CUR, ethyl acetate extracts from *T. chrysantha* (0.5 μg/mL) and *T. rosea* (1 µg/mL) and induction of oxidative stress with 0.6 mM H _2_O _2_. Kruskal Wallis * *p*<0.05. ALA: Alpha-lipoic acid; CUR: Curcumin.

Transcriptional regulation of antioxidant response genes against oxidative stress represents a defense against cell damage. In this study, we evaluated the expression of the gene coding for the antioxidant enzyme NQO1, which is involved in protection against oxidative stress. The level of expression of the *NQO1* gene was evaluated (qRT-PCR) and quantified using the 2 ^-ΔΔCt^ method. The results indicate that the ethyl acetate extract from both *T. chrysantha* and *T. rosea*, as well as the culture of HepG2 cells in the presence of H _2_O _2_, significantly increased the expression levels of *NQO1* after six hours of exposure ( *p*<0.05), compared to the controls ALA and CUR ( Figure 2). The relative expression levels of *NQO1* gene decreased significantly after 12 hours post-exposure.

## Discussion

Oxidative stress is important because of its relation with a wide variety of disorders associated with an increase in the levels of oxidative markers and damaged cellular components, such as Parkinson's disease, Alzheimer's disease, Huntington's disease, amyotrophic lateral sclerosis ^21^, premature aging, inflammatory diseases and cancer ^27^.

Plants are widely used as sources of antioxidants due to their phenolic compounds and ability to scavenge ROS and free radicals, which makes them among the most potent and therapeutically useful biocompounds ^28^. Some studies have evaluated the antioxidant activity in extracts obtained from *T. chrysantha* and *T. rosea* ^6, 29, 30^, demonstrating the potential of these plants in the search for new molecules with significant biological effects. Previous studies have also shown the potential anti-inflammatory activity of *T. chrysantha* and *T. rosea* ^6, 30^. Such activity can also be associated with and effect on Nrf2, a molecule that not only regulates oxidative/xenobiotic stress response, but also represses inflammation by opposing transcriptional upregulation of a number of pro-inflammatory cytokine genes ^31^. It is due to this that the antioxidant activity of the inner bark extracts obtained from *T. chrysantha* and *T. rosea* and its association with the activation-dependent translocation of Nrf2 to the nucleus and the induction in the expression of *NQO1* gene was evaluated.

The ethyl acetate extracts from both *T. chrysantha* and *T. rosea* displayed strong antioxidant activities due to their oxygen radical absorbance capacity, which could be related to the high total phenol content found with the Folin Ciocalteu method. This capacity could also be related to phenols previously reported in *T. rosea*, like gentisic acid ^32^ or phenols found in the same genus, such as α-tocopherol and γ-tocopherol ^33^. The results of the present study are in agreement with those previously reported for *T. rosea* ^6^. This is the first study involving the ethyl acetate extract from *T. chrysantha*. An evaluation of the scavenging hydroxyl radical capacity of the methanolic and aqueous extracts from *T. chrysantha* revealed significant scavenging of the hydroxyl radical (80 and 83%, respectively), and reductions in the production of the peroxyl radical ^30^.

Given that ethyl acetate extracts were the most active of those produced, and that they did not affect the viability of HepG2 cells, these extracts were used to evaluate effects on Nrf2 translocation and expression of antioxidant response genes. We compared basal Nrf2 levels in both the cytosol and the nucleus, as well as the changes associated with exposure to the extracts. As expected, after 4 hours of exposure of HepG2 cells to the extracts, Nrf2 translocate from the cytoplasm to the nucleus; however, this effect was more pronounced after exposure to ALA, CUR, and H _2_O _2_. Flavonoids found in the ethyl acetate extract of *T. rosea* ^6^ could be related to its ability to induce the activation and translocation of Nrf2, as they possibly have the same action mechanism as resveratrol, whose ability to activate Nrf2 translocation to the nucleus in astrocytes after 2.5 hours of exposure has been demonstrated previously ^34^. ALA may induce Nfr2 translocation to the nucleus after 1 hour of treatment ^35^. A recent study did show that pau d’arco (an extract from the inner bark of *T. impetiginosa*) has the capacity to activate and translocate Nrf2 to the nucleus via a MEK (MAPK/ERK kinase)-independent mechanism ^36^. The results show that the ethyl acetate extracts obtained from *T. chrysantha* and *T. rosea* did induce the nuclear translocation of Nrf2 in HepG2 cells. Therefore, the upregulated expression of the *NQO1* gene by the ethyl acetate extracts is due to the stabilization and nuclear accumulation of Nrf2.

Antioxidant activity through upregulated expression of *NQO1* gene has been reported for β-lapachone ^37^, a quinone that has previously been isolated from *T. chrysantha* ^38^. It is also possible that steroids found within ethyl acetate extracts of *T rosea* and *T. chrysantha* during the Lieberman-Burchard test could be responsible for the overexpression of *NQO1* gene, as that has been reported for steroids like 17β-estradiol on CCD841CoN cell line ^39^. The Nrf2 pathway is considered the most important in the cell for protection against oxidative stress, which is generated by an accumulation of ROS and/or electrophiles, leading to the oxidation of biomolecules, membrane damage, DNA adduct formation, and mutagenicity. All these changes lead to degeneration of tissues, premature aging, apoptotic cell death and the development of cancer ^13^.

Nrf2 is a major activator of the phase II antioxidant genes such as *SOD*, *CAT*, *GST*, *HO-1* and *NQO1* ^40^. Our results demonstrated that the ethyl acetate extracts from *T. chrysantha* and *T. rosea* increased the expression of *NQO1* in HepG2 cells after 6 hours of exposure compared to ALA and CUR, although only a significant difference was found for *T. chrysantha*. It has been shown that overexpression of Nrf2, by Nrf2-cDNA, upregulates the expression and induction of the *NQO1* gene in response to antioxidants and xenobiotics ^41^. In addition, Nrf2-null mice exhibited a marked decrease in *NQO1* expression and induction, indicating that Nrf2 plays an essential role in the *in vivo* regulation of *NQO1* in response to oxidative stress ^13^. *NQO1* overexpression is also considered a cytoprotective mechanism after exposure to toxic metals ^42^.

## Conclusion

The present study indicates that the ethyl acetate extracts obtained from the inner bark of *T. chrysantha* and *T. rosea* have promising antioxidant activity, as measured by the ORAC method. Both biocompounds have the ability to activate and translocate the Nrf2 transcription factor, inducing the expression of the *NQO1* gene. These results reinforce the importance of these plants in the search for new antioxidant molecules.

## Data availability

Underlying data is available from Open Science Framework.

OSF: Dataset 1. Nrf2-Mediated Antioxidant Activity Tabebuia. https://doi.org/10.17605/OSF.IO/9WZ86 ^43^

Licence: CC0 1.0 Universal (CC0 1.0) Public Domain Dedication
