# Supplementary material for: Nrf2-Mediated Antioxidant Activity of the inner bark extracts obtained from Tabebuia rosea (Bertol) DC and Tabebuia chrysantha (JACQ) G. Nicholson
Source: F1000Res. 2019 Feb 12;7:1937. Originally published 2018 Dec 16. [Version 2] doi: 10.12688/f1000research.17165.2 (PMC6347036; doi:10.12688/f1000research.17165.2)
Supplement: Peer review history

# Peer review: Referee response for version 1

**DOI:** 10.5256/f1000research.18765.r42265

**Published:** 2019-01-23

**Authors:** Silvia Quesada

**Affiliations:** 1 Department of Biochemistry, School of Medicine, University of Costa Rica (UCR), San Pedro, Montes Oca, Costa Rica

This article presents the preliminary phytochemical screening, total phenolic content and ORAC activity of different extracts obtained from the inner bark of *Tabebuia rosea* and *T. chrysantha*. The goal of the article is to study if the antioxidant activity of ethyl acetate extracts is mediated by Nrf2 nuclear translocation and its induction of antioxidant response gene (NQO1).

 The methodology as the results are clearly presented with enough detail to allow replication by others. Both ethyl acetate extracts present similar total phenolic content, however *T. rosea* ORAC activity is almost twice *T. chrysantha *activity. Also both extracts showed a similar preliminary phytochemical screening, with a little difference; *T. rosea* showed a positive FeCl _3_ test but a negative AlCl _3, _otherwise *T. chrysantha *presented a negative FeCl _3_ test but a positive AlCl _3_. Is this difference important?

 In the tests: Nrf2 activation and expression of NQO1, I think you should explain the purpose of using H _2_O _2_.

 Please find an annotated copy of the article here for further suggestions and comments.

I have read this submission. I believe that I have an appropriate level of expertise to confirm that it is of an acceptable scientific standard.

# Response

**Published:** 2019-02-05

**Authors:** Juan Carlos Sepulveda-Arias

**Affiliations:** Universidad Tecnológica de Pereira, Colombia

Dear Dr. Quesada. Thank you very much for your evaluation. We included in the manuscript that H _2_O _2_ was used as an oxidative stress inductor. We also included in the new version the suggestions you highlight in the PDF.

# Peer review: Referee response for version 1

**DOI:** 10.5256/f1000research.18765.r42264

**Published:** 2019-01-09

**Authors:** Jaime Martin‐Franco

**Affiliations:** 1 Grupo de investigación Química de Productos Naturales QPN, Departamento de Química, Universidad del Cauca, Popayán, Colombia

*Tabebuia* is the largest genus of the Bignoniaceae family and several bioactive compounds such as: naphthoquinones; quinones and phenols have been extracted so far, due to the traditional use of numerous species of this genus. Very few studies have evaluated the anti-oxidant activity of extracts obtained from both *T. rosea* and *T. chrysantha* and only one study has evaluated the effect of *T. impetiginosa* extracts on Nrf-2 activation and translocation.

 The study from Garzón-Castaño *et al.* describes for the first time the effect of the inner bark extracts from *T. rosea* and *T. chrysantha* on the Nrf-2-mediated antioxidant activity. The best antioxidant activity (evaluated using the ORAC Technique) was displayed by the ethyl acetate extracts from both species. This activity was clearly associated with the activation and translocation of Nrf-2 to the nucleus and the induction in the expression of the *NQO1* gene in HepG2 cells. This study contributes to the knowledge on the biological activity of plants from the genus *Tabebuia* that have not been studied extensively and highlight its importance on the search of new molecules with antioxidant activity.

 Finally, the research paper is very clear and presents results of great expectation.

I have read this submission. I believe that I have an appropriate level of expertise to confirm that it is of an acceptable scientific standard.
